# Cardiovascular status after Kawasaki disease in the UK

**DOI:** 10.1136/heartjnl-2015-307734

**Published:** 2015-08-27

**Authors:** V Shah, G Christov, T Mukasa, K S Brogan, A Wade, D Eleftheriou, M Levin, RM Tulloh, B Almeida, MJ Dillon, J Marek, N Klein, PA Brogan

**Affiliations:** 1 Infection, Inflammation and Rheumatology Section, UCL Institute of Child Health, London, UK; 2 Department of Paediatric Cardiology, Great Ormond Street Hospital NHS Foundation Trust, London, UK; 3 Department of Clinical Epidemiology, Nutrition and Biostatistics Section, UCL Institute of Child Health, London, UK; 4 Paediatric Infectious diseases group, Division of Medicine, Imperial College London, London, UK; 5 Department of Paediatric Cardiology, Bristol Royal Hospital for Children, Bristol, UK

## Abstract

**Objective:**

Kawasaki disease (KD) is an acute vasculitis that causes coronary artery aneurysms (CAA) in young children. Previous studies have emphasised poor long-term outcomes for those with severe CAA. Little is known about the fate of those without CAA or patients with regressed CAA. We aimed to study long-term cardiovascular status after KD by examining the relationship between coronary artery (CA) status, endothelial injury, systemic inflammatory markers, cardiovascular risk factors (CRF), pulse-wave velocity (PWV) and carotid intima media thickness (cIMT) after KD.

**Methods:**

Circulating endothelial cells (CECs), endothelial microparticles (EMPs), soluble cell-adhesion molecules cytokines, CRF, PWV and cIMT were compared between patients with KD and healthy controls (HC). CA status of the patients with KD was classified as CAA present (CAA+) or absent (CAA−) according to their worst-ever CA status. Data are median (range).

**Results:**

Ninety-two KD subjects were studied, aged 11.9 years (4.3–32.2), 8.3 years (1.0–30.7) from KD diagnosis. 54 (59%) were CAA−, and 38 (41%) were CAA+. There were 51 demographically similar HC. Patients with KD had higher CECs than HC (p=0.00003), most evident in the CAA+ group (p=0.00009), but also higher in the CAA− group than HC (p=0.0010). Patients with persistent CAA had the highest CECs, but even those with regressed CAA had higher CECs than HC (p=0.011). CD105 EMPs were also higher in the KD group versus HC (p=0.04), particularly in the CAA+ group (p=0.02), with similar findings for soluble vascular cell adhesion molecule 1 and soluble intercellular adhesion molecule 1. There was no difference in PWV, cIMT, CRF or in markers of systemic inflammation in the patients with KD (CAA+ or CAA−) compared with HC.

**Conclusions:**

Markers of endothelial injury persist for years after KD, including in a subset of patients without CAA.

## Introduction

Kawasaki disease (KD) is a self-limiting medium vessel vasculitis of unknown aetiology affecting 8.39/100 000 children under the age of five per year in the UK;^1^ 22.5/100 000 in white Californians, with higher incidence in Asian/Pacific Islanders (50.4/100 000) and 29.8/100 000 in the black population.^2^ The highest incidence is in Japanese: 243.1/100 000 aged 0–4 years in 2011 and 264.8 in 2012.^3^ Coronary artery aneurysms (CAA) occur in 15%–25% of untreated patients; 2%–3% of untreated cases die as a result of coronary vasculitis.^4^ Despite the effectiveness of intravenous immunoglobulin (IVIG),^4^ 20% of cases are IVIG resistant, and are at even higher risk of coronary complications. As more children with KD are advancing into adulthood, further studies are needed to improve our understanding of long-term cardiovascular sequelae.^4–7^ KD vasculopathy differs from atherosclerosis in several key characteristics.^8^ Histologically, the vasculopathy of KD is characterised by an acute, predominantly neutrophilic necrotising vasculitis targeting the luminal coronary endothelium. Subsequently, a later subacute/chronic vasculitis ensues, targeting the adventitia, with myofibroblastic proliferation causing progressive arterial stenoses over many years. Importantly, histological features of true atherosclerosis such as lipid-laden macrophages are usually absent.^8^

The prognosis for patients with giant (≥8 mm) CAA is extremely worrying, with 88% 30-year survival, 16% myocardial infarction rate and 59% requiring revascularisation within a 25-year follow-up.^9^ Common sense dictates that patients with persistent CAA require lifelong follow-up. It remains unclear, however, what long-term management is required for patients who never had CAA or in whom CAA have regressed. Although many patients with CAA undergo regression of aneurysmal dilatation, the coronaries remain abnormally thickened, and vessel-wall calcification is often detected.^6^ The long-term consequences of these changes remain largely unknown. The American Heart Association (AHA) have issued clinical guidelines for the long-term follow-up and management of patients with KD,^10^ recently adopted in the UK.^4^ These recommendations are not based on robust scientific data. To date, there has only been a single small study of late KD vascular outcome in the UK.^11^ Long-term studies to inform follow-up strategies for patients with KD in the UK are, therefore, required. We conducted the current study to address this unmet clinical need.

We hypothesised that chronic vasculitis persists years after the acute KD presentation, and is detectable using novel biomarkers of endothelial injury. We conducted a study to examine the relationship between coronary artery (CA) status, endothelial injury, systemic inflammatory markers, cardiovascular risk, pulse-wave velocity (PWV) and carotid intima media thickness (cIMT) years after KD.

## Methods

This was an observational comparative study, with ethical approval (08/H0713/80). All participants provided fully informed written consent; assent, where appropriate, was also obtained from children under the age of 16 years. After an overnight fast, subjects attended the Somers Clinical Research Facility at Great Ormond St Hospital NHS Foundation Trust (GOSH) between January 2009 and May 2013 for a single research assessment. Deidentified clinical data collated included age, sex, ethnicity, age at KD diagnosis, features of KD at presentation, treatments past and present, CA status, body mass index, blood pressure and smoking status.

### Patients with KD

Inclusion criteria were complete KD (≥12 months previously) as defined by AHA criteria, fever lasting at least 5 days plus four of five principal clinical criteria: (1) rash, (2) bilateral conjunctivitis without exudate, (3) inflammation of oral mucosa, (4) cervical lymphadenopathy and (5) extremity changes.^10^ Patients with atypical KD with fewer than four of the clinical features, but in the presence of CAA, were also eligible for inclusion.^4^ Exclusions were presence of any significant acute or chronic comorbidity, including intercurrent infection. Patients with KD were recruited from two main sources: (1) GOSH and (2) via advertisement by the Kawasaki Syndrome Support Group (http://www.kssg.org.uk). CA status from the original clinical presentation for each patient was ascertained from independent direct scrutiny of patients’ medical records by a senior vasculitis expert (PAB) and a senior paediatric cardiologist (GC); any discrepant cases were discussed to achieve consensus. All clinical and scientific studies were performed blind to the subject status, however.

#### Definition of CA status and IVIG resistance

The primary data analysis was performed according to the worst-ever CAA status of the patients with KD, defined at any stage over the disease course using recommended AHA criteria.^10^ Thus, CAA+ patients were those where any of the following three conditions were met: (1) z score of left anterior descending (LAD) or right CA (RCA) ≥2.5, (2) abnormal CAs as per Japanese Ministry of Health criteria (internal diameter >3 mm in children under the age of 5 years, >4 mm for children 5 years and above, internal diameter greater than 1.5 times the size of an adjacent segment or obvious irregularity of the CA lumen) or (3) z score for LAD or RCA 2–2.5 in the presence of at least two other suggestive features (perivascular brightness, lack of arterial tapering, decreased LV function, mitral regurgitation or pericardial effusion). CAA− patients did not have any of the above features at any stage of their disease.

CAA on the day of study were defined as CA internal diameter ≥2 SD above the mean for age-adjusted body surface area, calculated using the web tool http://:www.paramaterz.com,^12^ and included those with true CAA (internal diameter Z score >3, giant aneurysms as internal diameter >8 mm) and those with CA ectasia (internal vessel diameter Z score ≥2, but <3),^10^ or other obvious significant luminal abnormality.

For the purposes of a subgroup analysis of the CAA+ patients, subjects who still had echocardiographic evidence of CAA on the day of study were defined as persistent CAA; CAA+ patients who no longer had evidence of CAA were defined as regressed CAA.

IVIG resistance was defined as those who received more than one dose of IVIG or one dose of IVIG plus second-line or third-line treatment (other than aspirin or other non-steroidal anti-inflammatory drugs).^4^

### Controls

Age-similar (at least within 2 years) and sex-matched controls were recruited from healthy unaffected siblings of patients with KD. A pilot study comparing the preliminary data obtained from the first 19 such controls found no difference in any biomarker compared with other matched paediatric controls from previous studies from our group.

#### Conventional cardiovascular risk factor assessments

Echocardiography was performed and interpreted in all subjects by trained senior paediatric cardiologists (GC, TM and JM) who were blinded to the subject's status using a predefined protocol that assessed CA and ventricular dimensions, conventional parameters of systolic and diastolic function (fractional shortening, EF, mitral inflow, pulsed wave tissue Doppler imaging annular velocities) and ventricular myocardial deformation using speckle-tracking imaging^13^; 12-lead resting ECG and fasting lipids (total cholesterol, low-density lipoprotein (LDL), high-density lipoprotein (HDL), very low-density lipoprotein (VLDL) and triglycerides (TG)) were also assessed using routine methodologies (see online supplementary methods). Resting (minimum of 15 min) blood pressure was measured using an oscillometric manual sphygmomanometer (Greenlight 300; ACCOSON, Essex, UK).

#### Assessment of inflammatory indices

High-sensitivity C reactive protein (hs-CRP), serum amyloid A (SAA), tumour necrosis factor α, interleukin-1β, 6, 8 and 10, monocyte chemoattractant protein-1, vascular endothelial growth factor (VEGF), angiopoietin 1 and 2, soluble E-selectin, soluble intercellular adhesion molecule 1 (sICAM-1), soluble vascular cell adhesion molecule 1 (sVCAM-1), soluble P-selectin and thrombomodulin were assessed using a validated commercial multiarray detection system based on electrochemiluminescence technology (SECTOR Imager 2400; MesoScale Discovery) (see online supplementary methods).

#### Assessment of endothelial injury

##### Circulating endothelial cells

Circulating endothelial cells (CECs) were identified with CD146-immunomagnetic bead extraction based on an international consensus standardised protocol,^14^ ^15^ as described in the online supplementary methods.

##### Endothelial microparticles

Annexin V-positive endothelial microparticles (EMPs) expressing CD105, E-selectin, ICAM-1, VCAM-1, CD144, CD31, but negative for the platelet marker CD42a, were quantified from platelet poor plasma using a BD FACSArray flow cytometer as previously described by our group,^16^ ^17^ (see online supplementary methods).

#### Vascular stiffness and cIMT

Carotid-femoral and carotid-radial PWVs were assessed by a trained investigator (VS) using the Vicorder device (Skidmore Medical) as per manufacturer's instructions, and in accordance with AHA recommendations^18^ (see online supplementary methods). Measurement of cIMT was assessed by experienced vascular technicians using a standardised imaging protocol (see online supplementary methods).

### Statistical analysis

Sample size calculations were based on CECs (the primary outcome measure). Pilot data from the first 30 patients with KD suggested that CECs were not normally distributed. Natural logarithmic transformation to normality provided an SD of 0.9 for the healthy controls (n=19) and 1.2 for patients with KD, and suggested that 40 subjects in each group were required to detect a doubling of average CECs in each KD subgroup versus controls with 90% power, significance 0.05. Making adjustment for non-normality and the need to either use non-parametric tests or to transform prior to analysis, this number increased to 47 per group.^19^ Thus, we aimed to recruit 80–100 KD subjects and 50 healthy controls. Numeric data were expressed as median and range, and were compared between groups using the Kruskal–Wallis and Mann–Whitney tests. Fisher's exact test was used to compare categorical variables. All differences, including 95% CIs, were calculated. Spearman correlation coefficient was used to explore the relationship between parameters. p Values of <0.05 were considered significant for CECs; analysis of all the other indices was considered exploratory, and therefore, p values were not adjusted for multiple comparisons. Analysis of covariance was used to compare the slope of PWV versus age between the groups using linear regression. Multivariable linear regression was used to assess the relationship between CECs (dependent variable) and predictor variables: healthy control or KD, presence of CAA, male sex, age at diagnosis and at study assessment, IVIG resistance, length of follow-up from KD diagnosis and inflammatory status (hs-CRP or SAA). CECs were log-transformed, and hence, model coefficients were exponentiated to represent fold-increase in median CEC between groups. Statistical analyses were performed using GraphPad Prism V.4.0, SPSS V.22 and R V.3.1.2.

## Results

### Demographics

Of 150 invites sent out, 92 positive responses and agreement to participate in the study were returned, yielding 92 patients with KD: 54 CAA− and 38 CAA+, and 51 healthy controls. The reason why some patients did not respond to the study invite was not formally ascertained; however, there was no obvious bias in CAA status in those who failed to respond. There was no difference in any demographic parameter, body mass index, blood pressure or smoking status between the KD group and controls (table 1). While the CAA+ group was slightly younger, this did not reach statistical significance. The CAA+ group was also younger at KD onset than the CAA− group. There were no other significant demographic differences between the controls and the KD subgroups. Subjects in both groups were predominantly Caucasian. Sixteen per cent in the control group were non-Caucasian; their ethnicities were Asian (n=4) or Afro–Caribbean (n=3); and 17% of the KD group were non-Caucasian; their ethnicities were Asian (n=8), Afro–Caribbean (n=6) and Brazilian (n=1).

**Table 1:** Demographics of patients with KD and healthy controls

	Healthy controls N=51	KD N=92	KD vs controls p Value	CAA− N=54	CAA− vs controls p Value	CAA+ N=38	CAA+ vs controls p Value
Age at study (years, range)	13.5 (4.9, 30.3)	11.9 (4.3, 32.2)	0.51	13.4 (5.3, 32.2)	0.61	10.4 (4.3, 24.4)	0.051
Males (%)	53%	51%	0.86	25/54 46%	0.56	22/38 58%	0.67
Body mass index, kg/m^2^	20.2 (13.5, 35.5)	19.2 (11.2, 33.6)	0.97	21.1 (14.0, 33.6)	0.21	17.3 (11.2, 29.7)	0.13
Systolic BP	110 (90, 133)	110 (78, 140)	0.72	110 (81, 137)	0.50	100 (78, 140)	0.12
Diastolic BP	60 (47, 80)	60 (40, 95)	0.99	60 (43, 95)	0.90	60 (40, 80)	1.0
Caucasian	82%	79%	0.82	87%	0.78	71%	0.46
Non-Caucasian	16%	17%		11%		24%	
Ethnicity unknown	2%	5%		2%		5%	
Smoker (%)	0%	3%	0.55	4%	0.50	3%	0.43
Age at KD diagnosis (years)	–	4.9 (0.18, 11.3)	–	3.0 (0.3, 11.3)	–	0.9 (0.2, 8.8)	–
Time of study (years) after KD	–	8.3 (1.0, 30.7)	–	8.5 (2.0, 30.7)	–	7.8 (1.0, 23.5)	–

All data are median (range), unless otherwise specified.

BP, blood pressure; CAA, coronary artery aneurysms; KD, Kawasaki disease.

### KD clinical features

Eighty-three out of 92 (90%) had complete KD; the remaining nine cases (10%) had atypical KD. Seventy-five received IVIG (82%); eight (9%) did not receive IVIG; in seven cases, the KD treatment status was unknown. Forty-eight cases (64%) were IVIG resistant; 63% of the IVIG-resistant group developed CAA, and 15% of the non-IVIG-resistant group developed CAA (difference 48%, 95% CI 25% to 63%, p<0.0001). Table 2 summarises the CA status of the 92 patients with KD. Twenty-two of the KD group were currently receiving medication. For the CAA+ group, 12 were on low-dose aspirin alone; three on aspirin and warfarin; one on aspirin and clopidogrel; one on aspirin, carvedilol and lisinopril and two on clopidogrel alone. For the CAA− group, one patient was on warfarin (for deep vein thrombosis (DVT) that complicated the initial KD episode), and one was treated with verapamil (for Wolff–Parkinson–White syndrome). None of the healthy controls were receiving medication.

**Table 2:** Coronary artery status of the KD group (n=92)

CAA status of the KD group (n=92)	n (% of all KD patients)
*CAA+*	*38 (41)*
CAA >8 mm	7 (8)
Persistent CAA	7 (8)
Regressed CAA	0 (0)
CAA <8 mm	31 (34)
Persistent CAA	10 (11)
Regressed CAA	21 (23)
*CAA−*	*54 (59)*

CAA+: CAA defined at any stage after the initial KD episode (see Methods). CAA−: no evidence of coronary aneurysm at any stage. Persistent CAA (n=17, 19%): any CAA+ subject who still had echocardiographic evidence of CAA on the day of study (see Methods). Regressed CAA (n=21, 23%): CAA+ subjects whose aneurysms had regressed on echocardiography on the day of study. 6/38 of the CAA+ patients developed coronary stenosis; these included 4/7 of the patients with giant (>8 mm) CAA, three of whom required a revascularisation procedure.

CAA, coronary artery aneurysm; KD, Kawasaki disease.

### Routine laboratory indices

Table 3 summarises the routine laboratory indices between the KD and control groups. There was no significant difference in hs-CRP, SAA, fasting total cholesterol, LDL cholesterol, HDL cholesterol or TG between healthy controls and patients with KD, with or without CAA.

**Table 3:** Hs-CRP, SAA and fasting lipids

	Healthy controls Median (range)	All patients with KD Median (range)	KD vs controls p Value (95% CI of diff)	KD CAA− Median (range)	CAA− vs controls p Value (95% CI of diff)	KD CAA+ Median (range)	CAA+ vs controls p Value (95% CI of diff)	p Value (Kruskal–Wallis)*
Hs-CRP (mg/L)	0.25 (0.00, 69.40) (n=49)	0.47 (0.01, 84.98) (n=89)	p=0.25 (−0.05 to 0.29)	0.46 (0.01, 11.16) (n=52)	p=0.30 (−0.07 to 0.28)	0.60 (0.02, 84.98) (n=37)	p=0.35 (−0.08 to 0.44)	0.51
SAA (mg/L)	0.80 (0.08, 14.71) (n=49)	1.21 (0.07, 225.2) (n=89)	p=0.22 (−0.11 to 0.62)	1.28 (0.07, 46.00) (n=52)	p=0.35 (−0.19 to 0.67)	1.19 (0.13, 225.2) (n=37)	p=0.23 (−0.13 to 0.74)	0.44
Total cholesterol (mmol/L)	4.40 (2.70, 5.90) (n=47)	4.15 (2.50, 6.70) (n=84)	p=0.44 (−0.4 to 0.2)	4.30 (2.50, 6.70) (n=49)	p=0.93 (−0.30 to 0.30)	4.00 (2.60, 5.50) (n=35)	p=0.16 (−0.6 to 0.1)	0.25
LDL (mmol/L)	2.27 (0.70, 4.34) (n=47)	2.28 (0.93, 4.90) (n=84)	p=1.00 (−0.26 to 0.24)	2.46 (1.04, 4.90) (n=48)	p=0.51 (−0.2 to 0.4)	2.25 (0.93, 3.66) (n=35)	p=0.41 (−0.44 to 0.15)	0.30
HDL (mmol/L)	1.40 (0.40, 2.40) (n=47)	1.40 (0.70, 2.10) (n=83)	p=0.37 (−0.2 to 0.1)	1.40 (0.70, 2.10) (n=48)	p=0.38 (−0.2 to 0.1)	1.40 (0.90, 2.00) (n=35)	p=0.52 (−0.2 to 0.1)	0.65
TG (mmol/L)	0.77 (0.39, 3.34) (n=47)	0.81 (0.34, 2.66) (n=84)	p=0.74 (−0.09 to 0.14)	p=0.81 (0.43, 2.66) (n=49)	p=0.78 (−0.1 to 0.16)	p=0.81 (0.34, 1.57) (n=35)	p=0.77 (−0.12 to 0.16)	0.94

All values are median (range) unless otherwise specified. Number (n) of subjects in each group as specified per test due to missing data points.

*p Values from Kruskal–Wallis test comparing three groups: controls, CAA− and CAA+. 95% CI of diff, 95% CI of the difference of median.

CAA, coronary artery aneurysms; HDL, high-density lipoprotein; hs-CRP, high-sensitivity C reactive protein; KD, Kawasaki disease; LDL, low-density lipoprotein; SAA, serum amyloid A; TG, triglycerides.

### Circulating inflammatory indices

The results are summarised in table 4. VEGF was higher in the CAA+ group than controls. There was no significant difference between healthy controls and patients with KD, with or without CAA, in any other inflammatory parameter studied.

**Table 4:** Circulating cytokines and other inflammatory indices in the KD group and controls

	Healthy controls Median (range) N=49	All patients with KD Median (range) N=90	KD vs controls p Value (95% CI of diff)	KD CAA− Median (range) N=53	CAA− vs controls p Value (95% CI of diff)	KD CAA+ Median (range) N=37	CAA+ vs controls p Value (95% CI of diff)	p Value (Kruskal–Wallis)*
MCP-1 (pg/mL)	219 (112, 490)	214 (88, 858)	p=0.48 (−32 to 15)	208 (99, 456)	p=0.22 (−44 to 9)	229 (88, 858)	p=0.83 (−28 to 38)	0.36
Ang1 (pg/mL)	44 302 (6835, 62 986)	41 120 (3212, 96 865)	p=0.51 (−5945 to 2482)	41 220 (3212, 75 043)	p=0.81 (−5642 to 3779)	39 452 (14 711, 96 865)	p=0.32 (−8191 to 2312)	0.61
Ang2 (pg/mL)	2303 (1063, 7830)	2340 (821, 9270)	p=0.61 (−255 to 424)	2468 (821, 6373)	p=0.60 (−283 to 517)	2285 (1023, 9270)	p=0.76 (−371 to 441)	0.83
VEGF (pg/mL)	92 (18, 475)	117 (11, 1469)	p=0.13 (−6 to 45)	100 (22, 1469)	p=0.48 (−15 to 33)	141 (11, 888)	**p=0.04 (1 to 90)**	0.10
TM (pg/mL)	3.82 (2.09, 9.10)	3.80 (1.20, 8.39)	p=0.69 (−0.67 to 0.40)	3.69 (1.20, 7.00)	p=0.35 (−0.9 to 0.30)	4.00 (1.70, 8.39)	p=0.68 (−0.58 to 0.8)	0.41
IL-1β (pg/mL)	0.71 (0.04, 3.66)	0.62 (0, 16.21)	p=0.32 (−0.28 to 0.12)	0.65 (0, 16.21)	p=0.74 (−0.25 to 0.20)	0.56 (0, 2.58)	p=0.13 (−0.42 to 0.07)	0.29
IL-8 (pg/mL)	3.60 (1.56, 9.60)	4.01 (1.57, 13.30)	p=0.19 (−0.18 to 0.9)	4.14 (1.57, 9.30)	p=0.14 (−0.17 to 1.10)	3.63 (2.17, 13.30)	p=0.51 (−0.40 to 0.87)	0.34
TNFα (pg/mL)	8.52 (2.39, 21.20)	8.94 (2.01, 51.9)	p=0.42 (−0.93 to 2.20)	9.44 (3.26, 41.00)	p=0.35 (−0.90 to 2.62)	8.35 (2.01, 51.90)	p=0.70 (−1.7 to 2.32)	0.66
IL-10 (pg/mL)	3.16 (1.00, 30.40)	3.63 (0.79, 65.40)	p=0.78 (−0.68 to 0.85)	3.37 (0.79, 63.80)	p=0.66 (−1.02 to 0.60)	4.23 (0.80, 65.40)	p=0.26 (−0.47 to 1.80)	0.29
IL-6 (pg/mL)	1.80 (0.50, 6.30)	1.78 (0.26, 13.60)	p=0.58 (−0.43 to 0.27)	1.56 (0.67, 5.03)	p=0.14 (−0.59 to 0.10)	2.01 (0.26, 13.60)	p=0.39 (−0.26 to 0.69)	0.08
TF (pg/mL)	42.10 (9.20–81.30)	39.00 (7.70–74.50)	p=0.71 (−3.40 to 6.30)	42.10 (13.90–60.90)	p=0.49 (−3.40 to 7.50)	39.00 (7.70–74.50)	p=0.87 (−6.30 to 5.90)	0.67

All values are median (range) unless otherwise specified. Number (n) of subjects in each group who had the test performed.

*p Values from Kruskal–Wallis test comparing three groups: controls, CAA− and CAA+. 95% CI of diff: 95% CI of the difference of median. Statistically significant results are in bold.

Ang, angiopoietin; CAA, coronary artery aneurysms; IL, interleukin; KD, Kawasaki disease; MCP-1, monocyte chemoattractant protein 1; TF, tissue factor; TM, thrombomodulin; VEGF, vascular endothelial growth factor.

### Assessment of endothelial injury

#### Circulating endothelial cells

**Figure 1:**
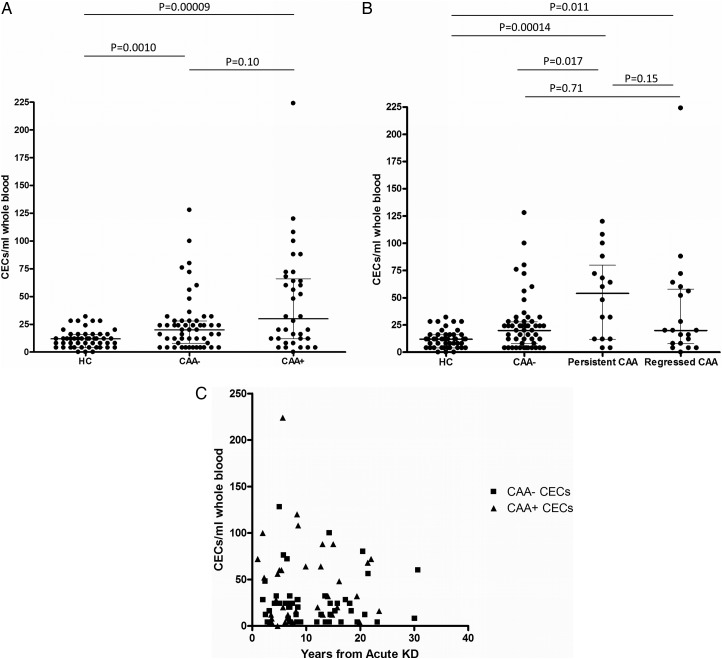
Circulating endothelial cells. (A) CECs were highest in the KD CAA+ group (CAA+ vs HC, 95% CI of difference in medians 8 to 44), but were not significantly higher than the CAA− group; CECs were also higher in the CAA− group versus HC (95% CI of difference in medians 4 to 12). (B) Patients with persistent CAA had even higher levels of CECs (persistent CAA vs HC, 95% CI of difference in medians 16 to 60) and higher versus CAA−. Those with regressed CAA also had high CECs (regressed CAA vs HC, 95% CI of difference in medians 0.000032 to 24) that were not significantly different from those with persistent CAA (95% CI of difference in medians −4 to 48) or the CAA− subjects. (C) There was no significant correlation between CECs and time from the acute KD episode to study: r=−0.13, 95% CI −0.23 to 0.44, p=0.36 for the CEC– group; r=0.12, 95% CI −0.40 to 0.44, p=0.50 for the CEC+ group. Horizontal lines represent median and IQR. CAA, coronary artery aneurysms; CECs, circulating endothelial cells; HC, healthy control; KD, Kawasaki disease.

Patients with KD had higher CECs than the healthy controls (table 5 and figure 1A). While the CAA+ group had the highest CECs, CAA− patients also had higher CECs compared with the controls. Subgroup analysis of the CAA+ group revealed patients with persistent CAA had the highest CECs, but those with regressed CAA also had significantly higher CECs than healthy controls (figure 1B). There was no significant correlation between CECs and time from the acute KD episode (figure 1C).

**Table 5:** Endothelial injury markers, platelet microparticles, arterial stiffness and carotid IMT in the KD group and controls

	Healthy controls Median (range)	All patients with KD Median (range)	KD vs controls p Value (95% CI of diff)	KD CAA− Median (range)	CAA− vs controls p Value (95% CI of diff)	KD CAA+ Median (range)	CAA+ vs controls p Value (95% CI of diff)	p Value Kruskal–Wallis*
CECs (cells/mL)	12 (0, 32) (n=49)	24 (0, 224) (n=89)	p=0.00003 (4 to 16)	20 (4, 128) (n=53)	p=0.0010 (4 to 12)	30 (0, 224) (n=36)	p=0.00009 (8 to 44)	p<0.0001
*Microparticles (×10^3^/mL) plasma*								
Total annexin V	990 (160, 6160)	970 (1190, 5410)	p=0.83 (−250 to 220)	850 (190, 5410)	p=0.42 (−360 to 160)	990 (230, 4360)	p=0.54 (−200 to 400)	0.37
CD54 (ICAM-1)	0.97 (0, 27.00)	0.87 (0, 18.00)	p=0.82 (−0.40 to 0.34)	0.85 (0, 18.00)	p=0.86 (−0.50 to 0.43)	1.10 (0, 9.11)	p=0.83 (−0.62 to 0.46)	0.97
CD62E (E-sel)	3.92 (0, 49.34)	2.87 (0, 22.37)	p=0.40 (−1.89 to 0.53)	3.13 (0, 22.37)	p=0.84 (1.75 to 0.96)	1.87 (0, 16.98)	p=0.16 (−2.97 to 0.19)	0.30
CD105	0 (0, 206.50)	1.60 (0, 186.80)	**p=0.04 (0.03 to 1.95)**	1.50 (0, 94.64)	p=0.14 (−0.02 to 1.67)	2.97 (0, 186.80)	**p=0.02 (0.01 to 6.00)**	0.07
CD62P (P-sel)	0 (0, 12.90)	0 (0, 15.17)	p=0.59 (−0.01 to 0.01)	0 (0, 15.17)	p=0.94 (−0.03 to 0.08)	0 (0, 8.62)	p=0.24 (−0.03 to 0.24)	0.41
CD144	0.20 (0, 12.36)	0.32 (0, 155.20)	p=0.42 (−0.02 to 0.29)	0.90 (0, 21.74)	p=0.24 (−0.004 to 0.71)	0 (0, 155.20)	p=1.0 (−0.02 to 0.02)	0.43
CD31	20.59 (0, 556.50)	14.18 (0, 981.00)	p=0.98 (−8.37 to 6.74)	13.62 (0, 981.00)	p=0.89 (−8.57 to 9.13)	17.63 (0, 258.30)	p=0.83 (−13.7 to 6.48)	0.93
VCAM-1 (CD106)	0 (0, 56.06)	0 (0, 110.50)	p=0.70 (−0.03 to 0.003)	0 (0, 5.76)	p=0.93 (−0.04 to 0.02)	0 (0, 110.50)	p=0.54 (−0.01 to 0.04)	0.79
CD42a (platelet MP)	24.93 (0, 930.70) (n=47)	14.04 (0, 586.50) (n=87)	p=0.26 (−15.88 to 2.02)	13.50 (0, 586.50) (n=52)	p=0.16 (−20.10 to 1.04)	13.61 (0, 509.90) (n=35)	p=0.66 (−16.25 to 6.74)	0.36
Soluble adhesion molecules (ng/mL)								
sICAM-1	251 (186, 504)	260 (131, 452)	p=0.58 (−16 to 30)	250 (153, 416)	p=0.46 (−31 to 16)	300 (131, 452)	**p=0.04 (1 to 59)**	**0.03**
sVCAM-1	408 (261, 1066)	465 (253, 880)	**p=0.007 (15 to 79)**	454 (253, 714)	p=0.08 (−4 to 66)	484 (274, 880)	**p=0.0022 (30 to 113)**	**0.006**
sE-Sel	13 (5, 62)	16 (5, 59)	p=0.31 (−1 to 4)	15 (5, 59)	p=0.49 (−2 to 4)	17 (5, 42)	p=0.27 (−2 to 5)	0.52
sP-Sel	70 (19, 262) (n=48)	82 (22, 204) (n=89)	p=0.10 (−2 to 23)	86 (23, 204) (n=53)	p=0.09 (−2 to 28)	77 (22, 139) (n=37)	p=0.28 (−7 to 22)	0.19
Carotid-radial PWV (m/s)	7.30 (4.70, 9.60) (n=51)	7.40 (4.60, 11.20) (n=91)	p=0.40 (−0.30 to 0.50)	7.50 (4.60, 11.20) (n=53)	p=0.25 (−0.20 to 0.70)	7.40 (4.90, 11.20) (n=38)	p=0.86 (−0.50 to 0.50)	0.46
Carotid-femoral PWV (m/s)	5.40 (4.00, 8.40) (n=51)	5.40 (3.80, 9.00) (n=91)	p=0.69 (−0.40 to 0.30)	5.70 (3.90, 9.00) (n=53)	p=0.58 (−0.30 to 0.60)	5.10 (3.80, 7.00) (n=38)	p=0.13 (−0.80 to 0.10)	0.15
Right CCA IMT (mm)	0.47 (0.40, 0.60) (n=42)	0.47 (0.39, 0.58) (n=78)	p=0.77 (−0.02 to 0.01)	0.47 (0.39, 0.58) (n=45)	p=0.59 (−0.02 to 0.01)	0.47 (0.39, 0.57) (n=33)	p=0.92 (−0.2 to 0.2)	0.75
Left CCA IMT (mm)	0.46 (0.41, 0.60) (n=40)	0.46 (0.37, 0.58) (n=76)	p=0.97 (−0.01 to 0.01)	0.47 (0.37, 0.53) (n=44)	p=0.99 (−0.02 to 0.02)	0.46 (0.39, 0.58) (n=32)	p=0.95 (−0.02 to 0.02)	1.00

All values are median (range) unless otherwise specified. Number (n) of subjects in each group as specified per test.

*p Values from Kruskal–Wallis test comparing three groups: controls, CAA− and CAA+ (CA status at initial disease presentation). 95% CI of diff: 95% CI of the difference of median. Statistically significant results are in bold.

CAA, coronary artery aneurysms; CCA, common carotid artery; CECs, circulating endothelial cells; E-Sel, E-selectin; ICAM, intercellular adhesion molecule; IMT, intima media thickness; KD, Kawasaki disease; P-Sel, P-selectin; PWV, pulse-wave velocity; s, soluble; VCAM, vascular cell adhesion molecule.

#### EMPs and soluble adhesion molecules

**Figure 2:**
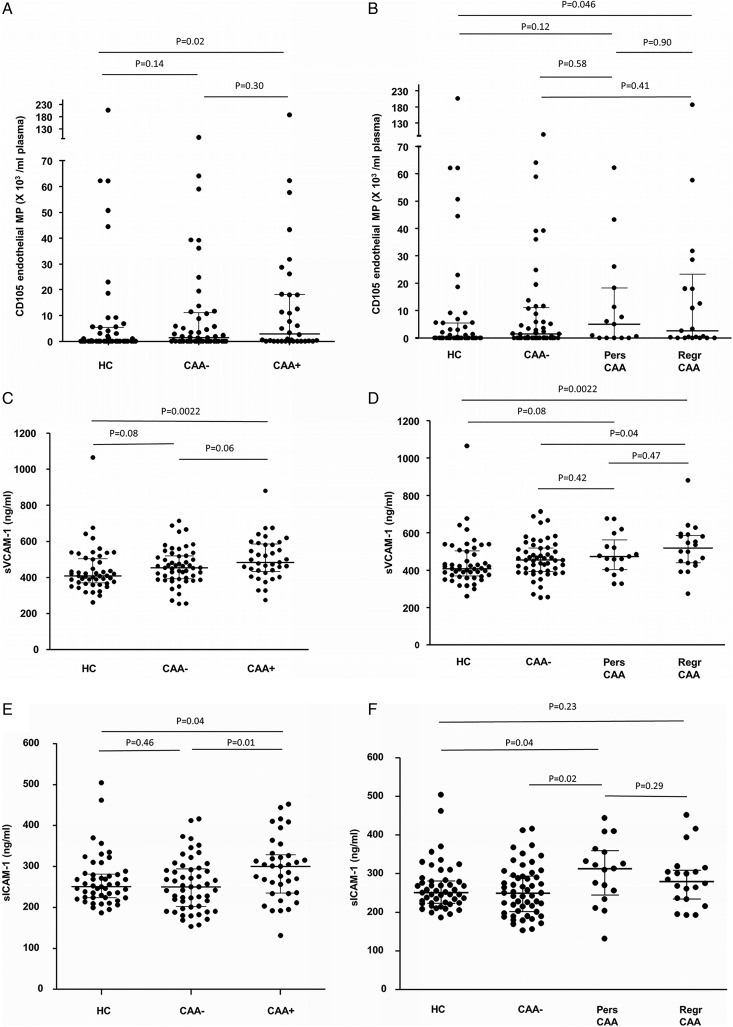
EMP and soluble adhesion molecules. (A) KD CAA+ patients had higher CD105 EMP than HC (95% CI of difference 0.01 to 6.00×10^3^/mL), but there was no significant difference when the CAA+ group was compared with CAA− patients. CAA− patients did not significantly differ from HC. (B) Patients with KD with persistent CAA had the highest CD105 EMP, although this did not reach statistical significance compared with controls (95% CI of difference in median −0.00005 to 6.04×10^3^/mL). Patients with KD with regressed CAA had significantly higher CD105 EMP (median 2.59×10^3^/mL) than HC (0.00×10^3^/mL) (95% CI of difference in median −0.00006 to 10.49), but not compared with the persistent CAA group (95% CI of difference in median −5.68 to 10.40) or the CAA− group. (C) sVCAM-1 was significantly higher in the CAA+ group versus HC, but this was not significantly higher when compared with the CAA− group.(D) sVCAM-1 was particularly high in those with regressed CAA (Regr CAA: vs HC (95% CI of difference in median 32.3 to 144.6 ng/mL) and vs CAA−), but was not different from those with persistent CAA (95% CI of difference in median −44.4 to 111.1 ng/mL). (E) sICAM-1 was higher in the KD CAA+ group (vs HC (95% CI of difference in median 1 to 59 ng/mL) and vs CAA−). (F) sICAM-1 was highest in those with persistent CAA (Pers CAA: vs HC (95% CI of difference in median 2 to 86 ng/mL) and higher than the CAA− group). There was no difference in sICAM-1 levels between the persistent and regressed CAA groups (95% CI of difference in median −76 to 31 ng/mL), and no significant difference between Regr CAA and controls (95% CI of difference in median −11 to 52 ng/mL). Horizontal lines represent median and IQR. CAA, coronary artery aneurysms; EMP, endothelial microparticles; HC, healthy control; KD, Kawasaki disease; Pers, persistent; Regr, regressed; sICAM, soluble intercellular adhesion molecule; sVCAM, soluble vascular cell adhesion molecule.

CD105 EMPs were higher in the KD group versus controls (table 5), particularly in the CAA+ group (figure 2A). Patients with persistent CAA had the highest CD105 EMPs, although patients with regressed CAA also had higher CD105 EMP than controls (figure 2B). sVCAM-1 was higher in the KD group versus controls (table 5), and even higher in the CAA+ group (table 5 and figure 2C). Patients with regressed CAA had the highest sVCAM-1 (figure 2D). sICAM-1 was higher in the CAA+ group versus the controls and the CAA− group (table 5 and figure 2E); patients with KD with persistent CAA had the highest sICAM-1 (figure 2F).

### Predictors of CEC counts

There was no significant association between CEC and current age, male sex, age at diagnosis, IVIG resistance, length of follow-up from KD episode, hs-CRP or SAA in univariable models (see online supplementary table S1). Both subject status (KD or healthy control) and presence of CAA were univariably significantly associated with CEC count. In the multivariable model, KD was associated with a 64% increase on average in CEC count (95% CI 12% to 140%, p=0.0001), and presence of CAA remained independently associated with a further 59% increase (95% CI 6% to 140%, p=0.026). After accounting for these two variables, none of the other factors were independently predictive of CEC count.

### Structural peripheral arterial parameters

**Figure 3:**
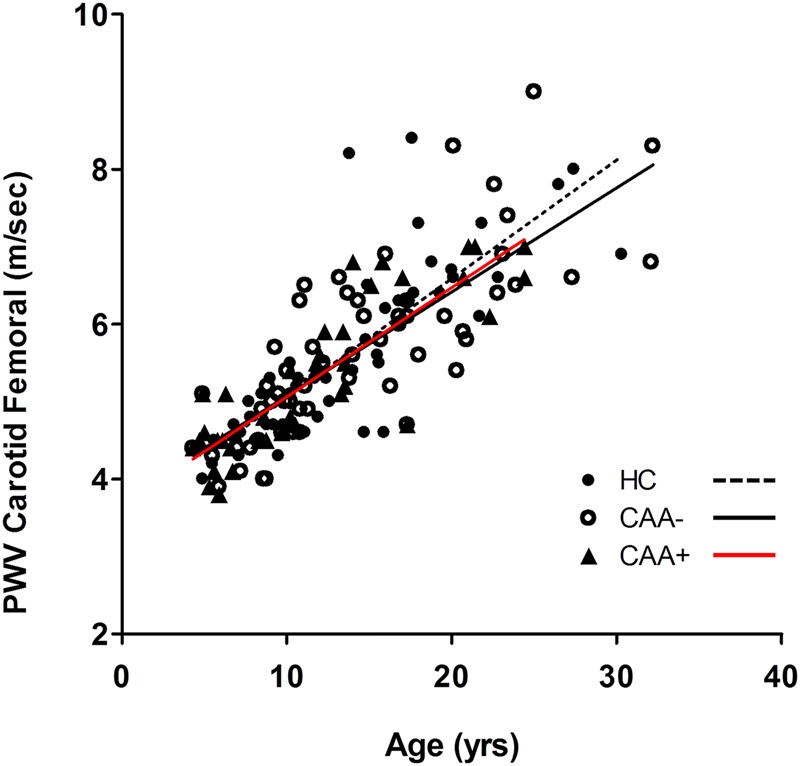
PWV versus age. There was a strong positive association between age and carotid-femoral PWV for all subject groups: r^2^=0.65 for controls, r^2^=0.62 for KD CAA− and r^2^=0.73 for KD CAA+; p<0.0001 for all. Analysis of covariance of the carotid-femoral PWV slope did not show any statistically significant difference from controls (dashed line) for KD CAA− patients (solid black line, p=0.87) or KD CAA+ patients (red line, p=0.66). CAA, coronary artery aneurysms; HC, healthy control; KD, Kawasaki disease; PWV, pulse-wave velocity.

There was a strong positive association between age and carotid-femoral PWV for all subject groups: r^2^=0.65 for controls, r^2^=0.62 for CAA− and r^2^=0.73 for CAA+; p<0.0001 for all (figure 3). There was, however, no difference in the PWV (table 5 and figure 3) or cIMT (table 5) between the controls and the patients with KD, with or without CAA.

## Discussion

The long-term cardiovascular outcome for paediatric survivors of KD is an important concern. It is known that the prognosis for patients with persistent CAA remains guarded, particularly for those with giant CAA since myocardial infarction occurs in 16%–31%.^9^ ^20^ Much less is known about the fate of those with regressed CAA or those who never developed CAA. We conducted a long-term follow-up study of KD in a UK-based population. Using a multimodal approach to the assessment of cardiovascular health, we observed elevation of markers of endothelial injury, a median of 8.3 (1.0–30.7) years after KD. CECs were significantly higher in KD than controls, and were highest in the CAA+ group, but also elevated in patients with regressed CAA and in the CAA− group (figure 1A, B). This observation was corroborated by further evidence of persistent markers of endothelial injury in KD versus controls from three other circulating indices: CD105 EMP, sVCAM-1 and sICAM-1, particularly in the CAA+ group (figure 2) and by the observation that VEGF was higher in the CAA+ group than controls. Patients with regressed CAA also had significantly higher circulating CD105 EMPs and sVCAM-1 (figures 2B, D). Despite this, we did not detect any evidence of altered peripheral arterial health (PWV and cIMT) in the patients with KD or any perturbation in conventional cardiovascular risk factors. These novel observations could have implications for long-term monitoring, follow-up and secondary prevention of cardiovascular events in survivors of KD.

CECs are mature cells that have become detached from the vessel wall, and are associated with vascular injury, particularly in diseases where endothelial injury is central to the pathogenesis, such as the systemic vasculitides.^16^ ^21^ Nakatani *et al*^22^ demonstrated high CECs in acute KD complicated by CAA, but also in patients without CAA. Yu *et al*^23^ demonstrated that these CECs expressed higher levels of inducible-nitric oxide synthase, mirroring findings in lesional coronary vasculitis observed at postmortem. These CECs carry surface-bound S100 and related proteins, including MRP-8/MRP-14,^24^ S100A12 25 and its receptor, RAGE (receptor for advanced glycation endproducts),^25^ ^26^ and their levels correlate with the severity of coronary vasculitis. These studies suggest that CECs detach from medium-sized arteries as a result of neutrophil-endothelial interactions mediated by S100 proteins. CECs appear highest in those with CAA, but are also elevated in patients with acute KD without CAA,^22^ and are higher in those with IVIG resistance.^26^ CECs may, therefore, be a good biomarker of acute coronary vasculitis.

Our study now suggests that CECs remain elevated for years after KD, including in some patients who never had any evidence of CAA. This finding aligns well with the observation that coronary endothelial dysfunction assessed using paradoxical vasoconstriction response to intracoronary acetylcholine challenge is known to persist for many years in patients with KD with aneurysms.^27^ ^28^ It is still unclear, however, how CECs relate to earlier studies that used flow-mediated dilatation (FMD) in the brachial artery. Dhillon *et al*^11^ observed that FMD of the brachial artery was markedly reduced in patients with KD compared with control subjects many years after the illness, even in patients without detectable early CA involvement. In contrast, McCrindle *et al*^29^ did not demonstrate any differences in brachial artery reactivity following KD. Brachial artery FMD, cIMT and PWV are unlikely to be reliable surrogates for assessing coronary vascular health after KD since these techniques were developed for the assessment of vascular health in atherosclerosis. The peripheral arteries are not typically affected in KD, and hence, these indices are not good surrogates for the study of late-KD coronary vasculopathy.

We did not demonstrate any evidence of systemic inflammation (table 4) or cardiovascular risk factors to account for the elevated CECs we observed. We suggest that our observations are consistent with an ongoing active subclinical vasculitis because there was no significant correlation between CECs and time from acute KD diagnosis (figure 1C), and we also consistently observed elevated soluble adhesion molecules (sVCAM-1 and sICAM-1) and CD105 EMP in the KD CAA+ group. We also observed significantly higher levels of VEGF in the CAA+ group. This latter observation could be explained by an attempt at ongoing endothelial repair/remodelling in patients with active subclinical vasculitis, as suggested by our previous studies of systemic vasculitis in children.^16^ It could also indicate an ongoing, and possibly permanent, disturbance in endothelial cell homoeostasis. Future studies examining the mechanisms driving this chronic endothelial injury and the prognostic relevance of this finding are now required. While we did not measure circulating S100 proteins or their expression on CECs, it is possible that this mechanism could account for the persistently high CEC levels.

Although we did (as expected) observe a strong positive relationship between PWV and age, there was no difference in patients with KD (with or without CAA) and healthy controls (figure 3). Similarly, we did not detect any difference in cIMT. These findings are in agreement with a recently published large study of cardiovascular health in 203 predominantly Caucasian North American patients with KD, a median of 11.6 years (1.2–26 years) after acute KD;^30^ the authors concluded that patients with KD without CAA or with CA ectasia could be reassured, and may not need specialised surveillance. Moreover, we did not observe any difference in lipid profile between patients with KD and controls again, in agreement with the study conducted by Selamet Tierney *et al*.^30^ While we cannot yet conclude that patients with high CECs after KD are at higher risk of cardiovascular events, our study now provides a scientific rationale for the AHA recommendation for follow-up even for those without CAA,^10^ since persistence of subclinical vasculitis in an important subset of patients is likely to act in concert with traditional cardiovascular risk factors to adversely influence long-term coronary prognosis. Since it is currently not possible to clinically distinguish those with elevated CECs from patients with KD as a whole, we suggest that all patients with KD (irrespective of CAA status) require long-term monitoring. In particular, the current practice of discharging those with ‘resolved’ aneurysms from care no longer seems prudent. Long-term surveillance and registries to follow these patients into adulthood to monitor outcomes are required.

### Limitations

Since our study assessed medical records from multiple centres going back over 30 years, it is possible that some patients with minor degrees of coronary vasculitis could have been misclassified, although medical records were assessed independently by two experienced clinicians, thus limiting any potential bias. Second, since our study was not a single-centre study, sequential detailed echocardiographic data were not available to us to study CAA regression rates and the potential influence of that on CEC counts, an area worthy of a further prospective study. Third, for practical reasons, we used healthy siblings of index KD cases as controls. As yet undefined genetic factors could, therefore, have influenced the cardiovascular status of these healthy sibling controls. We consider this to be unlikely, however, even more so because there was no signal of this in a pilot study comparing these controls with other healthy young controls from separate studies. Lastly, regarding peripheral arterial health, the median time after KD was relatively short, although there was no signal whatsoever that PWV (figure 3) or cIMT (table 5) deviated from controls even 20–30 years after KD.

## Conclusions

In conclusion, we have conducted a follow-up study of KD and found: (1) evidence of persistent endothelial injury years after KD in a subset of patients both with and without CAA and (2) no evidence of structural peripheral arterial disease. Future studies now need to validate the relevance of CECs as a potentially important prognostic biomarker of late-KD vasculopathy.

**Key messages**

**What is already known on this subject?**

- Kawasaki disease (KD) is a self-limiting medium vessel vasculitis of unknown aetiology, affecting predominantly children under the age of 5 years, resulting in coronary artery aneurysms (CAA) in approximately 25% of untreated patients. There is a need to identify patients at risk of late KD vasculopathy to inform clinical strategies for surveillance and prevention of late cardiovascular events.

**What might this study add?**

- This study examined circulating endothelial cells (CECs) years after KD. CECs were increased in patients with KD, were highest in those with CAA, but were also elevated in some patients without CAA, compatible with a state of persistent subclinical vasculitis years after the acute disease. In contrast, arterial stiffness, carotid intima media thickness and conventional cardiovascular risk factors were no different from controls.

**How might this impact on clinical practice?**

- The significance of patients having high CECs is unknown. Our study does, however, provide a scientific rationale for recommending lifelong follow-up of all patients with KD.

## Supplementary Material

Web supplement
