# Supplementary material for: Cardiovascular status after Kawasaki disease in the UK
Source: Heart. 2015 Aug 27;101(20):1646–55. doi: 10.1136/heartjnl-2015-307734 (PMC4621377; doi:10.1136/heartjnl-2015-307734)
Supplement: Web supplement [file heartjnl-2015-307734-s1.pdf]

Supplementary Methods and Results

Supplementary methods

Fasting lipid assay

Fasting lipids were measured using the VITROS Chemistry System (Ortho-Clinical Diagnostics, Raritan, NJ) from serum samples by multilayer film dry-slide chemistry with colorimetric detection.

Assessment of inflammatory indices

Circulating soluble markers of systemic and vascular inflammation were studied using a multi-parametric approach to explore potential relevant inflammatory pathways. High sensitivity C reactive protein (hs-CRP), serum amyloid A (SAA), tumour necrosis factor alpha (TNF-α), interleukin (IL)- 1β, 6, 8 and 10, monocyte chemoattractant protein-1 (MCP-1), vascular endothelial growth factor (VEGF), angiopoietin 1 and 2, soluble E-selectin (sE-sel), soluble intercellular adhesion molecule 1 (sICAM-1), soluble vascular cell adhesion molecule 1 (sVCAM-1), soluble P selectin (sP-Sel), and thrombomodulin (TM) were assessed using a multi-array detection system based on electro-chemiluminescence technology (SECTOR Imager 2400, MesoScale Discovery; see supplementary methods). In brief, this system uses multi-array plates fitted with multi-electrodes per well with each electrode being coated with a different catching antibody. The assay procedure then follows that of a classic sandwich ELISA with the analytes of interest captured on the relevant electrode. These captured analytes were then in turn detected by a secondary analyte-specific ruthenium-conjugated antibody, which is capable of emitting light after electrochemical stimulation. A particular advantage of this system is the ability to simultaneously measure different biomarkers in small (25ul or 50uL) serum or plasma samples. Tissue Factor (TF) was measured by sandwich enzyme immunoassay using a commercially available kit from R & D Systems, Europe Ltd, (Abingdon, UK).

Circulating endothelial cells

Venous blood (1 ml) collected into tubes containing EDTA was mixed with buffer (1 ml of phosphate buffered saline containing 0.1% bovine serum albumin and 0.6% sodium citrate) and 20 µl of Fc receptor–blocking reagent (Miltenyi Biotec) and incubated for 5 minutes at room temperature. Fifty microliters of a preparation of anti-CD146-coated immunomagnetic beads (clone S-endo-1; BioCytex and Dynal Biotech) was added, and the sample was incubated at 4°C for 30 minutes, with rotation. Bead-bound cells were separated using a magnet (MPC-L; Dynal Biotech) and washed 3 times with buffer. Cells were then resuspended in 100 µl of buffer containing 10 µl of a 2-mg/ml preparation of FITC-labeled Ulex europaeus lectin (Sigma-Aldrich) and incubated for 1 hour at room temperature in the dark. CECs in the sample were counted using a Nageotte chamber on a fluorescence microscope by an experienced scientist (VS) blinded to the study subject status. CECs were defined as Ulex bright cells that were >10 µm in size, with >5 magnetic beads attached.

Endothelial microparticles (EMPs)

Blood was collected in 3.2% buffered citrate and centrifuged at 5000 g for 5 min twice to obtain platelet-poor plasma (PPP). MPs were sedimented from 200µl of PPP after centrifugation at 17000g for 60 min and re-suspended in An V binding buffer (BD PharMingen, Oxford, United Kingdom) prior to incubating with conjugated fluorescent monoclonal proteins or antibodies labelled with fluorescein isothiocyanate [FITC], phycoerythrin [PE], or Allophycocyanin (APC): An V FITC (BD Oxford, United Kingdom), mouse (PE)-labeled anti-human CD62e (Clone 68-5H11, BD PharMingen Oxford, United Kingdom), mouse (PE)-labeled anti-human CD31 (clone WM59, BD Oxford, United Kingdom), mouse (PE)-labeled anti human 62P (clone AK-4 BD PharMingen Oxford, United Kingdom), mouse (PE)-labeled anti human CD105 (clone SN6 E-Bioscience Hatfield United Kingdom), mouse (PE)-labeled anti human CD54 (clone HA58 BD PharMingen Oxford, United Kingdom), mouse

(PE)-labeled anti human CD106 (clone BD 51-10C9 PharMingen Oxford, United Kingdom). Additional labeling with mouse anti-human CD42a-APC (clone GR-P Immunostep.com Salamanca Spain) to exclude MP of platelet-origin was conducted. Samples were analyzed with a FACS Array flow cytometer (BD).

Vascular stiffness and carotid intima media thickness

Carotid-femoral and carotid-radial pulse wave velocity (PWV) was assessed by a trained investigator (VS) using the Vicorder device (Skidmore Medical Limited) as per manufacturer instructions, and in accordance with American heart Association recommendations (23).

The technique most widely used to measure arterial stiffness is the determination of arterial pulse wave velocity (PWV). PWV is the speed of travel of the pulse along an arterial segment. Carotid-femoral PWV is a direct measurement, and it corresponds to the widely accepted propagative model of the arterial system. However, carotid-radial PWV may also provide other relevant information of arterial stiffness. For this study, PWV measurements were obtained using the Vicorder device (Skidmore Medical Devices) by placing a 100 mm wide blood pressure cuff around the upper thigh to measure the femoral pulse, and a 30mm partial cuff around the neck at the level of the carotid artery. High quality waveforms were recorded simultaneously for 3 seconds with the subject in the supine position, and the foot-to-foot transit time was determined using an in-built cross-correlation algorithm centered around the peak of the second derivative of pressure. For carotid to femoral PWV, path length was defined as the distance from the suprasternal notch to the middle of the thigh cuff as indicated by the manufacturer (mm). The measurement from the suprasternal notch to the umbilicus and then to the middle of the cuff was recorded, in addition. The distance from the suprasternal notch diagonally to the middle of the neck cuff was also recorded as a separate

measurement. For carotid to radial PWV, path length was defined from the distance from the suprasternal notch to the middle of the radial cuff.

Measurement of far wall carotid intima-media thickness (cIMT) with B-mode ultrasound is a non-invasive and reproducible technique for identifying and quantifying vascular disease and for evaluating cardiovascular risk. For this study, experienced vascular technicians carried out all cIMT measurements following a standardised imaging protocol (see acknowledgments). The Zonare ultrasound scanner (Zonare Medical System) with a high resolution probe, was used to image both the right (RCCA) and left (LCCA) common carotid arteries longitudinally 1cm proximal to the carotid bifurcation. Images were focussed on the posterior (far) wall of the artery and the zoom function was used to magnify the area. Ten second cineloops were recorded in DICOM format and downloaded for offline analysis. Three end-diastolic frames were selected and analysed for mean cIMT, defined as the interface between lumen-intima and media-adventitia, for both right and left carotid arteries using an automated carotid analyser (Carotid Analyser, M.I.A). The images were analysed by accredited readers and the mean of both the left and right-sided readings was used for the analysis.

Supplementary Results

Supplemental Table 1: Predictors of CEC counts

Variables	Unadjusted fold increase in median CEC (95%CI)	P value
Presence of CAA	1.37 (1.40,2.92)	**0.0002**
Subject status (healthy control/KD)	1.38(1.40,2.74)	**0.0001**
Current age (years)	0.99 (0.70,1.21)	0.559
Males	0.87 (0.55,1.07)	0.119
Serum amyloid A	1.01 (0.97,1.14)	0.244
Hs-CRP	1.01 (0.99,1.07)	0.777
Age at diagnosis (years)	0.96 (0.87,1.05)	0.364
Years of follow up post KD	1.01 (0.97,1.0)	0.959
IVIG resistance	1.15 (1.76,1.53)	0.333

Supplemental Table 1 legend: Unadjusted univariable analysis for fold increase in median circulating endothelial cell (CEC) count in association with presence of CAA, subject status, current age, sex (male), serum amyloid A, hs-CRP, age at diagnosis of KD, years of follow up post KD, and IVIG resistance.
